# 异基因造血干细胞移植患者脑脊液EB病毒检测的临床意义

**DOI:** 10.3760/cma.j.issn.0253-2727.2023.09.006

**Published:** 2023-09

**Authors:** 云 何, 瑞 马, 慧芳 王, 晓冬 莫, 圆圆 张, 萌 吕, 晨华 闫, 昱 王, 晓辉 张, 兰平 许, 开彦 刘, 晓军 黄, 于谦 孙

**Affiliations:** 北京大学人民医院血液科，北京大学血液病研究所，国家血液系统疾病临床医学研究中心，造血干细胞移植治疗血液病北京市重点实验室，北京 100044 Peking University People's Hospital, Peking University Institute of Hematology, National Clinical Research Center for Hematologic Disease, Beijing Key Laboratory of Hematopoietic Stem Cell Transplantation, Beijing 100044, China

**Keywords:** 造血干细胞移植, EB病毒, 脑脊液, Hematopoietic stem cell transplantation, Epstein-Barr virus, cerebrospinal fluid (CSF)

## Abstract

**目的:**

分析异基因造血干细胞移植患者脑脊液EB病毒检出情况及其临床意义。

**方法:**

2017年1月至2022年6月，1100例在北京大学人民医院接受异基因造血干细胞移植的血液病患者因出现中枢神经系统症状送检脑脊液病毒检测，其中19例脑脊液EB病毒检测阳性，回顾性分析其临床特征、治疗及预后。

**结果:**

19例脑脊液EB病毒阳性患者中男12例，女7例，年龄<18岁5例，≥18岁12例，中位年龄27（5～58）岁。急性髓系白血病7例，急性淋巴细胞白血病8例，再生障碍性贫血2例，霍奇金淋巴瘤1例，噬血细胞综合征1例。全部19例患者均为单倍体造血干细胞移植，其中二次移植1例。19例患者均有神经系统症状（头痛、头晕、抽搐或癫痫发作），其中13例患者伴发热。10例头颅影像学检查未见异常。19例患者中6例诊断为EB病毒相关中枢神经系统疾病，中位诊断时间为移植后50（22～363）d。9例（47.3％）患者外周血EB病毒检测阳性，均予以利妥昔单抗静脉滴注治疗（其中2例患者行腰椎穿刺鞘内注射给药），治疗后7例患者血EB病毒转为阴性。19例患者中2例复发，2例死于EB病毒感染，2例死于其他原因。

**结论:**

异基因造血干细胞移植患者出现中枢神经系统症状时，应进行EB病毒检测；脑脊液EB病毒检测阳性提示存在EB病毒感染，但不能作为确诊依据。

## Abstract (en)

**Objective:**

To analyze the detection rate, clinical significance, and prognosis of Epstein-Barr virus (EBV) in the cerebrospinal fluid (CSF) of patients following allogeneic hematopoietic stem cell transplantation.

**Methods:**

A retrospective analysis was performed on 1100 patients who underwent the CSF virus test after allogeneic hematopoietic stem cell transplantation in Peking University People's Hospital between January 2017 and June 2022. Among them, 19 patients were screened positive for EBV in their CSF, and their clinical characteristics, treatment, and prognosis were analyzed.

**Results:**

Among 19 patients with EBV-positive cerebrospinal fluid, 12 were male and 7 were female, with 5 patients aged <18 years and 12 aged ≥18 years, with a median age of 27 (5–58) years old. There were 7 cases of acute myeloid leukemia, 8 of acute lymphocytic leukemia, 2 of aplastic anemia, 1 of Hodgkin's lymphoma, and 1 of hemophagocytic syndrome. All 19 patients underwent haploid hematopoietic stem cell transplantation, including 1 secondary transplant. Nineteen patients had neurological symptoms (headache, dizziness, convulsions, or seizures), of which 13 had fever. Ten cases showed no abnormalities in cranial imaging examination. Among the 19 patients, 6 were diagnosed with EB virus-related central nervous system diseases, with a median diagnosis time of 50 (22–363) days after transplantation. In 9 (47.3％) patients, EBV was detected in their peripheral blood, and they were treated with intravenous infusion of rituximab (including two patients who underwent lumbar puncture and intrathecal injection of rituximab). After treatment, EBV was not detected in seven patients. Among the 19 patients, 2 died from EBV infection and 2 from other causes.

**Conclusion:**

In patients who exhibited central nervous system symptoms after allogeneic hematopoietic stem cell transplantation, EBV should be screened as a potential pathogen. EBV detected in the CSF may indicate an infection; however, it does not confirm the diagnosis.

EB病毒（EBV）是一种常见的疱疹病毒，90％以上的成年人有EBV感染史[1]–[2]，感染后病情呈自限性，随后EBV长期潜伏在体内，在免疫功能受损时再次激活。异基因造血干细胞移植（allo-HSCT）患者由于免疫功能严重低下，EBV再激活的发生率为0.1％～63％[3]–[4]，多数患者表现为EBV血症，严重者可引起移植后淋巴增殖性疾病（PTLD）、肺炎、肠炎等EBV终末器官疾病，也可累及中枢神经系统。

中枢神经系统EBV感染极其罕见，可以表现为脑炎、脑膜炎、脊髓炎、血管炎、PTLD、感染后脱髓鞘病变等不同临床类型[5]。尽管脑组织病理检查是中枢神经系统EBV感染诊断的金标准，但很少有患者能够接受脑组织活检。因此，中枢神经系统EBV感染的诊断需根据临床表现、影像学改变以及病原学检测综合判定并排除其他病因。

使用PCR方法检测脑脊液EBV尽管应用广泛，但目前并不足以作为确定诊断的依据。由于外周血单核细胞中存在EBV-DNA，脑脊液PCR检测可能会存在假阳性[6]。既往文献显示，在免疫功能正常人群中，21.4％的脑脊液EBV阳性患者最终被诊断为非感染性疾病[7]。在艾滋病患者中，脑脊液EBV检测的阳性率为1.9％～36.0％[8]–[13]。但在allo-HSCT患者脑脊液EBV检测的意义尚无报道。本研究中，我们对19例脑脊液EBV检测阳性allo-HSCT患者的临床特征及预后进行回顾性分析。

## 病例及方法

1. 病例：2017年1月至2022年6月期间，1100例在北京大学人民医院接受allo-HSCT的患者因出现中枢神经系统症状送检脑脊液病毒检测，其中19例（1.73％）EBV检测阳性，回顾性分析其临床特征、治疗及预后。

2. 移植预处理及移植物抗宿主反应病（GVHD）预防方案：19例患者均为单倍体造血干细胞移植，除1例二次移植患者预处理方案为氟达拉滨/环磷酰胺（Flu-Cy）外，其余患者接受改良白消安/环磷酰胺（Bu-Cy）联合兔抗人胸腺细胞球蛋白清髓预处理方案。GVHD预防方案为环孢素A（CsA）、霉酚酸酯（MMF）联合甲氨蝶呤（MTX）。

3. 定义：EBV血症定义为连续2次检出血EBV≥500拷贝/ml[14]，EBV相关PTLD分为临床诊断（Probable PTLD）和确诊（Proven PTLD）[15]–[16]。EBV脑炎/脑脊髓炎诊断标准参见文献[17]–[18]。

4. EBV检测及处理：我中心所有allo-HSCT患者移植后100 d内常规每周行1～2次EBV检测。移植后患者发生中枢神经系统症状时行腰椎穿刺，进行脑脊液EBV检查。EBV阳性患者处理：①抢先治疗：若患者为EBV血症，可密切监测，条件允许情况下减停免疫抑制剂；具有EBV病/EBV相关PTLD高危因素（T细胞去除、供受者EBV血清学不合、脐血干细胞移植、HLA配型不合、脾切除、二次移植、重度急性GVHD或需免疫抑制治疗的慢性GVHD、间充质干细胞治疗）[15]或EBV持续升高达10 000拷贝/ml以上的患者，可应用利妥昔单抗直至EBV检测转阴。②EBV病/EBV相关PTLD的治疗：若EBV阳性（>1 000拷贝/ml）且合并其他临床表现（发热、淋巴结肿大、低氧血症等），尽早给予利妥昔单抗治疗（375 mg/m^2^每周1次），不必等待淋巴结活检结果。

5. 脑脊液检测：移植后出现中枢神经系统症状的患者进行腰椎穿刺，测颅内压，脑脊液标本送检常规、生化、浓缩查瘤细胞、白血病免疫分型检测及PCR病原检测。EBV检测包括定量检测和定性检测（分为阳性、弱阳性、可疑阳性），脑脊液病原检测包括结核/非结核分枝杆菌、副流感、腺病毒、呼吸道相关病原［呼吸道合胞病毒、人冠状病毒（HCoV-229E、HCoV-HKU1、HCoV-NL63、HCoV-OC43）、人鼻病毒、支原体、衣原体、嗜肺军团菌］、疱疹病毒［单纯疱疹病毒（HSV）、人类疱疹病毒6型（HHV-6）、人类疱疹病毒8型（HHV-8）、水痘带状疱疹病毒（VZV）、EBV和巨细胞病毒（CMV）］、肠道病毒、细小病毒等。

6. 影像学检查：所有出现中枢神经系统症状的患者均进行头部CT或磁共振成像（MRI）检查。

7. 随访：所有患者均通过门诊或电话随访至2022年11月30日，统计患者EBV情况，评估移植相关事件发生率及总生存时间（OS）。

## 结果

一、病例基本特征

19例脑脊液EBV检测阳性患者中男12例，女7例，<18岁5例，≥18岁12例，中位年龄27（5～58）岁。急性髓系白血病7例，急性淋巴细胞白血病8例，再生障碍性贫血2例，霍奇金淋巴瘤1例，噬血细胞综合征1例。全部19例患者均为单倍体造血干细胞移植，其中二次移植1例。19例患者均为清髓预处理方案（Bu/Cy 15例，TBI/Cy 3例，Cy/Flu 1例）。GVHD预防方案为MMF+CsA+MTX，其中5例患者为母亲及旁系供者，在MMF+CsA+MTX基础上加用移植后环磷酰胺（Cy 14.5 mg/kg，+3 d及+5 d各1次）。移植物：骨髓联合外周血干细胞7例，外周血干细胞12例。输注MNC中位数为9.9（7.0～17.2）×10^8^/kg，CD34^+^细胞中位数为3.2（1.2～11.8）×10^6^/kg。所有患者均获得粒细胞植活，中位植入时间为13（10～23）d；19例患者中17例血小板植活，中位植入时间为13（10～33）d。6例患者发生Ⅰ～Ⅱ度急性GVHD，2例患者发生Ⅲ/Ⅳ度急性GVHD，1例发生慢性GVHD。7例（36.8％）患者发生CMV感染。

二、EBV检出情况

所有19例患者均有中枢神经系统症状（头疼5例，头晕5例，抽搐或癫痫发作5例），13例患者有发热。19例脑脊液EBV阳性的患者中，10例影像学检查未见异常。脑脊液检查WBC升高者占26.3％（5/19），总蛋白升高者占47.3％（9/19），总蛋白0.53（0.14～1.53）g/L，葡萄糖3.66（0.79～7.18）mmol/L，氯化物120.8（116.0～145.9）mmol/L。19例患者均同时进行了多种病原检测，其中1例HSV阳性，2例HHV-6阳性，1例脑脊液培养为脑膜炎奈瑟菌。19例患者脑脊液EBV定性检测（PCR法）均为阳性、弱阳性或可疑阳性，6例行EBV定量检测。所有19例患者均进行了外周血EBV检测，其中9例（47.3％）阳性。

三、治疗及结局

19例患者中6例诊断为EBV相关中枢神经系统疾病（EBV脑炎/脑膜炎4例，EBV相关弥漫大B细胞淋巴瘤2例），中位诊断时间为移植后50（22～363）d。19例患者中有2例诊断为中枢神经系统白血病，2例考虑为可逆性后部脑病综合征，1例诊断为癫痫，1例诊断为HHV6脑炎，1例诊断脑膜奈瑟菌感染。

19例患者中9例血标本EBV检测阳性，均予以静脉利妥昔单抗2～4剂治疗；除2例患者外，其余7例患者血EBV检测转阴。6例诊断为EBV相关中枢神经系统疾病的患者中4例仅行1次腰穿，2例患者除静脉使用利妥昔单抗375 mg/m^2^外，还进行了利妥昔单抗鞘内注射，脑脊液EBV分别于37 d及42 d转阴。以上9例血EBV检测阳性患者中，1例因继发噬血细胞综合征死亡，2例死于原发病复发，1例死于粒细胞缺乏伴感染，余5例存活。

## 讨论

EBV是中枢神经系统感染的潜在病原。脑脊液中检出EBV高度提示EBV是可能的致病原。有研究显示，在780例免疫功能正常的人群中，42例（5.4％）EBV检测阳性，33例（78.6％）诊断为EBV感染（脑膜炎1/3，脑炎2/3）[7]。艾滋病患者中，脑脊液EBV检测阳性率为1.9％～36％[8]–[13]；allo-HSCT患者脑脊液EBV相关中枢神经系统疾病的发生率为6.97％（12/172）[18]。在本研究中，1100例移植后出现中枢神经系统症状的患者脑脊液EBV检测阳性率为1.73％（19/1100），6例考虑为EBV相关中枢神经系统病变，提示对于免疫功能低下人群，伴有神经系统症状时，EBV应作为潜在的病原进行检测。

allo-HSCT后EBV相关脑炎文献报道不多。Liu等[18]报道3年期间172例allo-HSCT患者中12例发生EBV相关中枢神经系统疾病，发生率为（8.6±2.4）％，中位发生时间为移植后49（22～184）d，4例患者头MRI异常，12例患者中9例接受了利妥昔单抗为基础的治疗，最终5例患者存活。柯鹏等[17]报道allo-HSCT患者EBV脑炎发生率为0.7％（7/998），中位发生时间为移植后63（10～136）d，4例头MRI检查阳性。本中心1100例移植后出现中枢神经系统症状的患者中，脑脊液EBV检测阳性率为1.72％（19/1100），EBV相关中枢神经系统疾病的发生率为0.54％（6/1100），中位诊断时间为移植后50（22～363）d，其中4例诊断为EBV脑炎/脑膜炎，2例为EBV相关弥漫大B细胞淋巴瘤，有3例患者头影像学检查异常，和既往文献报道相似。

尽管脑脊液检出EBV是诊断中枢神经系统EBV感染的重要依据，但EBV的检出不足以作为中枢神经系统EBV感染的确诊依据。在本组病例中，13例患者脑脊液EBV检测阳性但并未诊断中枢神经系统EBV感染，考虑检出的低拷贝数EBV可能是来自于潜伏感染的淋巴细胞。国际诊断共识推荐血清学方法（包括抗EBV衣壳抗原IgM/IgG及抗核抗原）联合PCR方法用于诊断中枢神经系统EBV感染[6]。

EBV共感染是另一个值得关注的现象。文献报道脑脊液EBV检测阳性的患者中，22.7％～28％可同时检测到其他病原[19]–[21]。在此类患者中，脑脊液PCR检出EBV时应谨慎解释。

中枢神经系统EBV感染患者的预后较差，目前缺乏有效治疗手段。条件允许情况下，脑脊液EBV-DNA阳性的住院患者应考虑减少免疫抑制剂使用。利妥昔单抗在治疗EBV相关淋巴增殖性疾病方面的疗效已得到充分证实[16],[22]–[23]，然而，利妥昔单抗穿越血脑屏障能力较差，利妥昔单抗静脉给药在EBV相关脑炎中能否发挥作用尚不清楚。抗病毒药物和静脉注射免疫球蛋白的作用机制也尚未明确。静脉更昔洛韦、阿昔洛韦及糖皮质激素也有报道[24]–[25]，但病例数较少。EBV脑炎患者预后较差，鞘内注射给药可能是一种可行的办法[17],[26]–[27]。

本研究病例数较少且为单臂回顾性分析，多数病例为EBV定性检测，未能进一步分析EBV拷贝数的意义，无法区分脑脊液EBV检测阳性是EBV激活还是脑脊液中白细胞升高所致。因此，我们认为allo-HSCT患者脑脊液EBV检测阳性的意义在于提示存在EBV感染，但不能作为确诊依据。
